# Emerging Techniques in the Treatment of Conjunctival Melanoma

**DOI:** 10.1007/s40135-025-00334-9

**Published:** 2025-06-13

**Authors:** Wendy J. Li, Nandini Venkateswaran, Michael Antonietti, Sana Qureshi, Anat Galor, Carol L. Karp

**Affiliations:** 1 Department of Ophthalmology, Bascom Palmer Eye Institute, 900 NW 17 thStreet, Miami, FL 33136 USA; 2 Massachusetts Eye and Ear Infirmary, Harvard Medical School, Boston, MA USA; 3 Department of Ophthalmology, Miami Veterans Hospital, Miami, FL USA

**Keywords:** Conjunctival melanoma, Melanocytic lesions, Primary acquired melanosis, Surgical excision, Brachytherapy, Immunotherapy

## Abstract

**Purpose of review:**

In this article, we summarize the pathogenesis, diagnostic challenges, current management, and emerging therapeutic strategies for conjunctival melanoma (CM).

**Recent findings:**

CM is a deadly disease with rising global incidence. Key mutations, including BRAF, NF1, and TERT, play crucial roles in CM pathogenesis and may serve as targets for therapy. Advanced imaging and histopathological analysis have improved early detection and prognostic assessment. Treatment depends on tumor stage and includes surgical excision with adjuvant chemotherapy, radiotherapy, or orbital exenteration in advanced cases. Systemic workup is always indicated. Immune checkpoint inhibitors, successful in cutaneous melanoma, show promise in CM based off early studies, although more research is needed to confirm their benefit.

**Summary:**

CM remains a significant clinical challenge and requires a multidisciplinary approach to diagnosis and treatment. Incorporating genetic profiling and targeted therapies is crucial to improving patient outcomes.

## Introduction

Conjunctival melanoma (CM) is a rare but potentially life-threatening malignancy arising from melanocytes in the basal layer of the conjunctival epithelium. This extraocular tumor has gained increasing attention in recent decades due to its rising incidence, mirroring trends observed in cutaneous melanoma [1–3]. With a global incidence of 0.1 to 0.9 cases per million person-years, CM represents a significant challenge in ocular oncology [1, 2, 4–6].

As the second most prevalent conjunctival malignancy after ocular surface squamous neoplasia (OSSN), CM accounts for 2% of all ocular malignancies and 5–10% of ocular melanomas [1, 7–9]. Despite its rarity compared to cutaneous melanoma, which occurs 360 to 900 times more frequently, CM’s impact on patient morbidity and mortality is substantial [10, 11].

CM can affect various parts of the conjunctiva, including the bulbar and palpebral regions, as well as the caruncle. Its distinct etiology and genetic profile set it apart from other ocular melanomas, such as choroidal melanoma, necessitating a unique approach to diagnosis, treatment, and management.

CM has significant morbidity and mortality and a high rate of local recurrence and metastasis. Risk factors for recurrence include older age, history of prior surgery, lack of pigmentation, and advanced American Joint Committee on Cancer (AJCC) T category [8, 12]. Local recurrence rates at 5 and 10 years range from 26%−60% and 31%−66%, while metastasis rates at the same intervals range from 17%−52% and 27%−57%, respectively [13–17]. Studies have reported 5- and 10-year mortality rates of 17–29% and 30–50%, respectively [6, 18–21].

Given its increasing incidence and significant impact on patients’ lives, a comprehensive understanding of CM's epidemiology, pathogenesis, clinical presentation, and management strategies is crucial for improving patient care and survival. This review aims to provide an up-to-date overview of CM, focusing on recent advances in diagnosis, treatment, and ongoing research efforts in this challenging field of ocular oncology.

## Risk Factors and Prognosis

CM arises from three primary sources: primary acquired melanosis (PAM) with atypia (42%–74%), de novo occurrence (11%–38%), and less commonly, from pre-existing conjunctival nevi (2%–33%) [13, 16, 17, 22–25]. The prevalence of CM increases with age, peaking in the seventh and eighth decades of life [6, 26–28].

The role of ultraviolet (UV) radiation in CM development, while well-established in cutaneous melanoma, has been a subject of debate [29, 30]. However, epidemiological studies have revealed a correlation between decreasing latitude (towards the equator) and increasing CM incidence, suggesting a potential link to UV exposure [31–34]. This association is further supported by the observation of increased CM incidence on sun-exposed areas, such as the bulbar conjunctiva and caruncle [4, 35]. Molecular findings in CM patients, including C to T mutation signatures typical of UV-induced damage, provide additional evidence for UV radiation's role in CM pathogenesis [31, 36]. UV radiation's impact on CM risk is complex, involving both UVA and UVB. While UVB directly damages DNA and primarily affects the superficial epidermis, UVA penetrates deeper into the dermal stroma and generates reactive oxygen species [37, 38]. Both types of UV radiation may contribute to CM development through different mechanisms.

Prognosis in CM is influenced by patient age, AJCC classification, orbital invasion, and type of initial surgery [39, 40]. Patients older than 70 years of age generally present with larger tumors and greater rates of recurrence. Individuals with higher T categories have an increased 10-year rate of visual acuity loss, local recurrence or new tumors, need for exenteration, and locoregional lymph node metastasis [41]. Each increase in the AJCC T category is linked to a higher risk of metastasis [12]. Patients with orbital invasion have significantly higher 10-year rates of exenteration, distant metastasis, and mortality [12, 42]. Early detection and the initial surgery are crucial in preventing tumor seeding, recurrence, and metastasis. Tumor origin and Fitzpatrick skin type have not been shown to impact patient outcomes [39].

Tumor location is also a prognostic indicator in CM. Lesions in the palpebral and forniceal conjunctiva, plica semilunaris, and caruncle are associated with less favorable outcomes compared to limbal and epibulbar tumors, which show lower rates of recurrence and distant metastasis [13, 16, 25, 43]. Other factors affecting prognosis include growth pattern, cell type, and origin. A nodular growth pattern is associated with a poorer prognosis [43]. On histopathology, mixed cell tumors are associated with a mortality rate three times higher than that of spindle tumors [25]. CM arising de novo has been associated with an increased risk of local recurrence, distant metastasis, and death [13, 44].

CM primarily metastasizes through lymphatic spread, initially to the parotid and preauricular nodes, followed by the submandibular and cervical nodes [16, 45]. Distant metastasis, which can occur independently of regional disease, commonly affects the lungs, liver, and brain [45]. Lymphatic invasion of tumor cells is associated with a four-fold increase in mortality [25]. Tumor-associated lymphangiogenesis is associated with higher rates of local recurrence, distant metastasis, and melanoma-related death [44].

Understanding these risk factors is crucial for accurate prognosis and effective management of CM, highlighting the need for comprehensive assessment and tailored treatment strategies for each patient.

## Tumor Genetics and Immunology

Cancer development is a complex process driven by a series of genetic and genomic alterations that enable cancer cells to evade tumor suppression, sustain proliferative signaling, and resist cell death. A crucial aspect of this process is the interplay between proto-oncogenes and tumor suppressor genes. Proto-oncogenes regulate positive cell growth and survival, and their overactivation through mutations can promote cancer cell growth. In contrast, tumor suppressor genes regulate cell division and replication; when mutated, they fail to check cellular growth, contributing to cancer progression. Key genes involved in CM biology include *BRAF, NF1, NRAS,* and *KIT* [36, 39, 46, 47]*.*

In CM, two primary oncogenic pathways become overactive: the mitogen-activated protein kinase (MAPK) pathway and the PI3 K-AKT pathway [46, 48]. These pathways drive cell survival and proliferation, making them critical targets for modern treatment approaches such as *BRAF* and *MEK* inhibitors [49–51].

The genetic landscape of CM is characterized by several key mutations.* BRAF* mutations are observed in approximately one-third of CM patients, with the most common mutation being V600E, accounting for approximately 80% of *BRAF* mutations [35]. These mutations are early events in tumor growth and are more frequently seen in younger male patients with lesions located in sun-exposed areas, mixed or absent pigmentation, or a nevus origin [35]. Mutations in the *NF1* gene are observed in about one-third of patients and result in MAPK pathway activation [52]. The *TERT* gene, which is involved in telomerase production, is mutated in 32–43% of primary CM cases and correlates with metastatic disease [53, 54]. *TERT* plays a key role in tumorigenesis by ensuring chromosomal stability and allowing cells to avoid senescence [55, 56]. *NRAS* mutations are present in 0–18% of CM patients and lead to the continuous activation of both MAPK and PI3 K signaling pathways; these mutations are generally mutually exclusive with *BRAF* mutations. [57] *KIT* mutations are rare, occurring in less than 1% of patients with CM [57, 58]. Some tumors also exhibit *ATRX* mutations, which are involved in telomere maintenance [8].

Additionally, chromosomal copy number alterations (CNA) contribute to the tumorigenesis of CM. The most frequently reported CNA is a 6p amplification, which is observed in more than 60% of patients [59]. Furthermore, the loss of 10q has been linked to the development of metastasis [59]. As research progresses, insights into these genetic alterations may lead to more personalized treatment strategies for CM.

A summary of tumor genetics and targeted therapies can be found in Table 1.

**Table 1:** Summary of key genetic alterations and immune checkpoint targets in CM, their effects on cellular signaling, potential targeted therapies, and the frequency of each mutation. The MAPK pathway is a common target, with BRAF, NRAS, and NF1 mutations collectively affecting a significant proportion of CM cases. Targeted therapies for these alterations primarily focus on inhibiting components of the MAPK pathway. Less frequent mutations in KIT and ATRX also present potential therapeutic targets

Conjunctival Melanoma Genetics and Targeted Therapies			
Genetics/Target	Mechanism	Conjunctival melanoma targeted treatment	Mutation Frequency in Conjunctival Melanoma
BRAF	MAPK pathway activation	*BRAF inhibitors* Dabrafenib Encorafenib Selumetinib Vemurafenib	31–35% [8, 35, 60]
NF-1	MAPK pathway and mTOR pathway activation	*MAPK inhibitors* *mTOR inhibitors*	39% [8]
NRAS	MAPK and PI3 K-AKT pathway activation	*RAS inhibitors*	11–26% [8, 60]
TERT	Telomere maintenance	*telomerase inhibitors (experimental)*	41% [61]
ATRX	Telomere maintenance	No specific inhibitors	25% [8]
KIT	Tyrosine kinase signaling	Imatinib	3–4% [8, 60]
MEK	MEK 1 and MEK 2 proteins	*MEK inhibitors* Binimetinib Cobimetinib Trametinib	*N/A*
mTOR	PI3 K/AKT/mTOR pathway signaling	*mTOR inhibitor* Dactolisib	*N/A*
PD-1	T-cell inhibition	Pembrolizumab Nivolumab	*N/A*
CTLA-4	T-cell inhibition	Ipilimumab	*N/A*

## Clinical Diagnosis

The diagnosis of CM involves a comprehensive approach that combines detailed clinical examination with advanced imaging techniques. A thorough patient history is crucial, including information on age, symptoms, sun exposure, lesion evolution, prior cancers, and a review of previous photographs. This information provides valuable context for the subsequent physical examination, which should be meticulous and include lymph node palpation along with careful inspection of the ocular surface, everted eyelids, and adjacent skin.

**Fig. 1:**
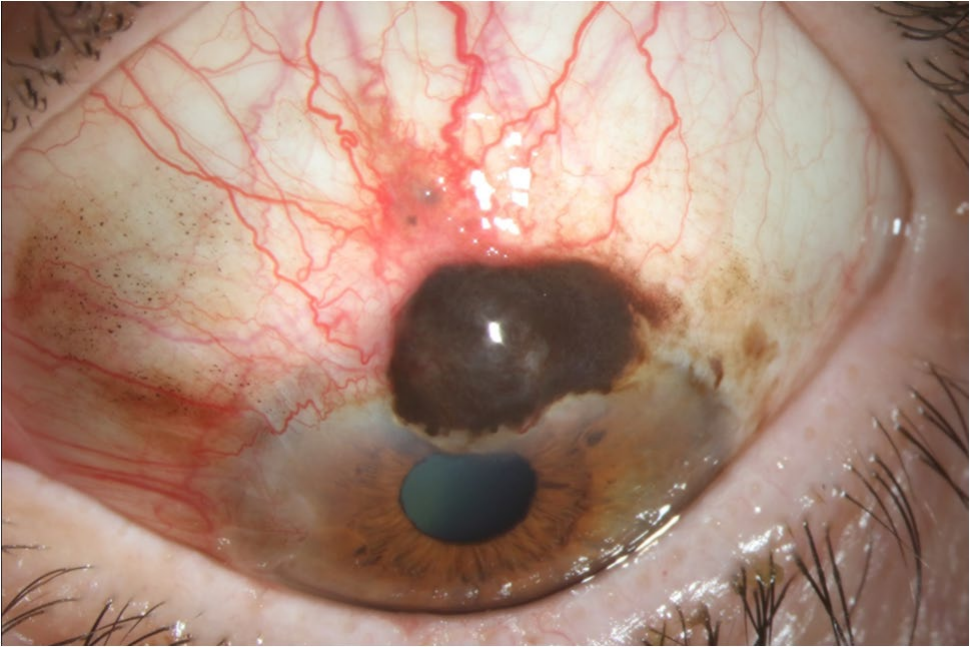
Slit-lamp photograph of conjunctival melanoma in a 60-year-old female. Slit lamp photography of the left eye demonstrates an elevated pigmented lesion at the limbus and over the superior cornea. Note the adjacent areas of pigmentation on the cornea and conjunctiva. Wide-margin excision with double freeze cryotherapy confirmed the diagnosis of malignant conjunctival melanoma

Slit lamp biomicroscopy is the cornerstone of the examination, allowing for detailed visualization of the ocular surface. CM typically presents as a unilateral, pigmented lesion involving the bulbar, forniceal, palpebral, and limbal conjunctiva. It is often immobile and vascular, appearing either flat or nodular with heterogeneous pigmentation (Fig. 1). Flat lesions may increase in thickness over time.

During the slit lamp examination, the examiner should meticulously assess the ocular surface for signs of pigment, nodularity, and feeder vessels, ensuring to examine areas like the fornix and tarsal conjunctiva by everting both the upper and lower eyelids. The presence of pigment, especially on the tarsal conjunctiva, in White patients should raise suspicion for CM. It is important to note that CM lesions can be amelanotic in 15–19% of cases or exhibit variable pigmentation and vascularity, which can complicate diagnosis [13, 17, 39, 62, 63].

Documentation is crucial. Slit lamp photographs should be taken to record the lesion's size and extent. Advanced imaging techniques such as anterior segment optical coherence tomography (AS-OCT) can provide further diagnostic information. AS-OCT images typically reveal a hyperreflective subepithelial lesion with normal to slightly thickened epithelium and variable hyperreflectivity of the basal epithelium. Hyperreflectivity of the epithelium is suggestive of involvement of the epithelium with atypical melanocytes. Additionally, a dilated fundus examination should be conducted to assess the internal surfaces of the eye. After CM is suspected based on clinical examination and imaging, excisional biopsy should be performed.

## Histopathologic Diagnosis

The definitive diagnosis of CM requires histopathologic analysis of a biopsy specimen, performed by an experienced pathologist. To facilitate accurate measurement of tumor thickness, histological sections should be cut perpendicular to the epithelial surface. Specimens are frequently bleached to remove melanin granules, which can obscure cellular morphology and structural details, as well as interfere with immunohistochemical analyses by disrupting antibody-antigen interactions [64].

CM tumors are characterized by four distinct types of atypical melanocytes: spindle cells, balloon cells, small polyhedral cells, and round epithelioid cells [15, 65]. Each type exhibits unique characteristics. Spindle cells are elongated with hyperchromatic nuclei and eosinophilic nucleoli [66]. Balloon cells appear larger with centrally placed nuclei and contain numerous clear vacuoles in the cytoplasm, giving the nuclei a scalloped appearance [67]. Small polyhedral cells, true to their name, are polygonal with clear cytoplasm and uniformly staining nuclei [68]. Round epithelioid cells, as the name suggests, are round with abundant eosinophilic cytoplasm and marked nuclear pleomorphism [65]. It is important to note that these cellular types are not exclusive to CM and can also be seen in other conditions such as conjunctival nevi and PAM with atypia, which is characterized by epithelioid cells [69]. The diagnosis of CM relies on correlating clinical observations with specific histological features, including pagetoid spread, radial growth of intraepithelial components, bandlike inflammation at the basal layer, significant mitotic activity, reduced maturation of basal cells, and invasion beyond the basement membrane layer of the sclera or rarely through Bowman's layer in the cornea [65].

The prognosis for CM is influenced by a complex interplay of several critical factors, including specific histological characteristics, tumor thickness, and the presence of genetic mutations. Histologically, lesions primarily made up of spindle cells are often associated with a better prognosis [25]. In contrast, the presence of pagetoid growth, over five mitotic figures per high power field, and an absence of an inflammatory response typically suggest a less favorable outcome [65]. The pathologist’s report is instrumental for informing treatment strategies, providing key details about the tumor's thickness and whether the surgical margins—both lateral and deep—are adequate [15].

Tumor thickness and ulceration are key prognostic indicators and are associated with increased risk of nodal metastasis, systemic metastasis, and death [12, 70–73]. The risk of distant metastasis is lowest for patients with tumors ≤ 1 mm thick and highest for patients with tumors > 4 mm thick. However, tumor thickness ≤ 2 and > 2 mm only have a borderline significant association with distant metastasis [74]. The presence of ulceration significantly worsens disease-specific survival, and a high mitotic rate is strongly associated with increased mortality [74].

Mutational profiling of the biopsy tissue is crucial for determining the presence of genetic mutations that may inform treatment decisions and impact prognosis. Specific mutations, including those in *BRAF* and *NRAS*, may be risk factors for recurrence, distant metastases, and shorter survival in CM patients [75, 76]. In addition, the presence of uveal melanoma-related hotspot mutations (including *BAP1*, *SF3B1*, and *GNAQ/11*) in CM has been linked to advanced disease and increased risk for metastasis and death [46]. Recent studies have also demonstrated an association between the presence of a *TERT* mutation and the development of metastasis and shorter metastasis-free survival [77]. Although *KIT* mutations are targetable with c-KIT inhibitors, their presence has not been shown to predict survival in CM.

In summary, the prognosis of CM is influenced by multiple factors, including histological cell type, tumor thickness, presence of ulceration, mitotic rate, and specific genetic mutations, all of which contribute to determining the potential course of disease, prognosis, and treatment targets. Histopathologic diagnosis remains an essential tool in this process, providing critical information that cannot be obtained through imaging or other diagnostic methods alone.

## Treatment of Early-stage Conjunctival Melanoma

The management of early-stage CM, particularly PAM with atypia, requires a nuanced approach based on several factors**.** PAM, also referred to as conjunctival melanocytic intraepithelial neoplasia (C-MIN), is an acquired area of flat, conjunctival pigmentation that usually occurs in middle-aged White patients [78]. It can be categorized as benign (PAM without atypia) or cancerous (PAM with atypia). PAM with atypia is the main risk factor for CM, with between 57–76% of all CMs arising from PAM [17, 79]. The treatment strategy for PAM is primarily determined by the lesion's degree of atypia, size, and location.

The histological degree of atypia in PAM is classified as mild, moderate, or severe, with each level carrying an increasing risk of progression to malignant melanoma. Mild atypia is characterized by atypical melanocytes confined to the basal layer of the epithelium, while severe atypia involves atypical melanocytes extending into the superficial non-basal portion of the epithelium in a pagetoid fashion [22]. Cases of PAM with no or mild atypia almost never transform into melanoma, whereas 13–46% of cases with severe atypia progress to melanoma [22, 80]. PAM without atypia, as confirmed by histopathological examination, does not require immediate intervention. However, careful observation and regular follow-ups are essential to monitor for any potential changes or progression. Patients with lesions suspicious for PAM should be promptly referred to a center with ocular oncology expertise, since early identification and referral can improve outcomes [21].

Newly discovered PAM is managed based on size. Small lesions measuring less than 2 clock hours (5 mm) and flat without associated vascularity may be closely observed [22]. In contrast, larger lesions between 2 to 6 clock hours typically warrant full excision with cryotherapy applied to the edges [81]. A personal or family history of cutaneous melanomas are other risk factors that might push toward excision of PAM, even in small lesions. Surgery is further indicated if the lesion shows growth, multifocality, or asymmetry compared to the contralateral eye [22]. If the lesion demonstrates even minimal nodularity, melanoma should be suspected and the lesion removed [82].

Large lesions that are not candidates for full surgical excision due to the risk of limbal stem cell deficiency or scarring may undergo map biopsies [81]. When map biopsies reveal PAM with atypia, further treatment is recommended. Treatment options include wide excision, cryotherapy, topical chemotherapy, or a combination of these approaches. Tumor location and extent are the main factors used to determine appropriate treatment modalities in PAM [22, 83].

### Surgical Excision

PAM with atypia is characterized as melanoma in situ, where atypical melanocytes are confined within the epithelium without breaching the basement membrane. In contrast, early CM exhibits initial deep invasion, with malignant cells penetrating beyond the epithelium into the underlying stroma. The cornerstone of treatment for localized disease is surgical excision using a ‘no-touch technique’, followed by double freeze/slow thaw cryotherapy. The ‘no touch technique’ described by Shields et al. aims to minimize tumor cell seeding, dissemination, and recurrence by removing the tumor en bloc without directly touching the mass and manipulating only surrounding normal tissue [84]. For CM, a wide excision with 3 to 4 mm margins along with removal of Tenon’s capsule is performed. Lamellar dissection of the scleral base (scleroconjunctivectomy) is typically performed if the lesion is deeper with scleral involvement [84]. Corneal involvement is addressed through manual removal or alcohol epitheliectomy. The corneal component is gently dried followed by direct application of absolute alcohol (ethyl alcohol) to the affected area using a cellulose sponge for 20–60 s [84]. The corneal lesion should then be removed and placed on paper to allow easy finding of the specimen. Cryotherapy can also be performed at the limbal margin. It is important to perform corneal removal without a disruption in Bowman’s layer to prevent any future corneal stromal involvement. During excision, use of a balanced salt solution should be avoided to prevent dissemination of tumor cells onto unaffected areas.

The excised lesion should be flattened out and placed on a cardboard bed. The paper should be labelled with the surgical pen on the surgical table, indicating the superior, inferior, or temporal margin. This is then re-traced once off the sterile field using pencil so that the markings do not disappear in formalin. Thinner lesions often adhere nicely to the paper after 60 s, but thicker lesions will need a suture to mark the margins. The specimen is preserved in 10% formalin and sent for histopathologic analysis. An accompanying history and drawing of the lesion and its orientation on the globe should be provided to the pathologist.

Following surgical excision, conjunctival reconstruction can be performed with primary closure for small lesions or by using an amniotic membrane graft [85]. For lesions involving the fornix, tarsal conjunctiva, or eyelid margin, a lamellar eyelid resection with an amniotic membrane graft may be required. Adequate conjunctival reconstruction is critical to avoid forniceal foreshortening or limitations in extraocular motility. While mucus membrane grafting is an excellent way to reconstruct fornixes, it is important to first ensure clear margins, as such grafts may hinder visibility for detecting recurrences. An oculoplastic surgeon should perform eyelid resection and reconstruction as needed. Post-operatively, symblepharon rings for two weeks may help maintain the fornix anatomy.

Cryotherapy may be performed after surgical resection to treat the conjunctival margins in a double freeze-slow thaw technique. This destroys any remaining tumor cells by inducing cellular ischemia through microvascular disruption [84]. During cryosurgery, a cryoprobe tip creates an ice ball 2 mm in size at the conjunctiva, 1 mm for episcleral tissues and the corneoscleral limbus, and 0.5 mm for the cornea. The ice ball is then allowed to slowly thaw and is refrozen in the same location [86]. Most surgeons will use cryotherapy as an adjunctive treatment following surgical excision in CM [45, 82]. While cryotherapy can be used as solo therapy when treating PAM with atypia or recurrent positive margins after surgical excision, it should not be used as primary therapy for CM due to high recurrence rates reported in the literature [87]. For deeper lesions, surgeons may also apply absolute alcohol or mitomycin C (MMC) to the scleral base; cryotherapy is often avoided on the scleral base because it may cause scleral melting. Neoadjuvant therapy with topical MMC may also reduce the pigmentation and size of the PAM lesion prior to resection [88].

Surgical excision is the primary treatment for CM, but adjuvant therapy with topical agents or radiotherapy may be indicated on a case-by-case basis [15, 84]. When margins are positive for invasive melanoma after excision, especially in those with high-risk features such as caruncular or palpebral location, repeat surgery should be considered. In cases of residual intraepithelial disease after surgical excision, adjuvant topical therapies may be employed. Given the significant risk of local recurrence and potential for metastasis, it is important to consider combining surgical excision with adjunctive cryotherapy, chemotherapy, immunotherapy, and/or brachytherapy [62, 89–92]. Topical chemotherapy with MMC is the medical treatment of choice when surgical margins demonstrate residual PAM with atypia or intraepithelial disease postoperatively [93–98]. For patients with positive deep margins, plaque brachytherapy is also an effective treatment [99, 100].

### Topical Chemotherapy

Topical chemotherapy plays a supportive role in the management of CM, serving as an adjunct rather than primary therapy due to its limited standalone efficacy. Its application is particularly valuable in specific scenarios following surgical excision and cryotherapy. These include cases with evidence of residual intraepithelial disease or diffuse or multifocal PAM. Topical chemotherapy as an adjuvant therapy has the advantage of treating the entire ocular surface, especially in eyes with poorly defined tumor margins. Additionally, it can treat diffuse corneal lesions or multifocal lesions where complete surgical resection might compromise limbal stem cell function or significantly reduce native conjunctival tissue. The most well-studied and commonly used topical agent is MMC [88, 98, 101, 102]. Some studies have demonstrated the effectiveness of immunotherapy agents such as interferon-alfa-2b (IFN-a2b) [103–105]. No significant evidence supports the efficacy of 5-fluorouracil for PAM. The drawbacks of using topical chemotherapy to treat PAM lesions include challenges with patient adherence, ocular surface toxicity, and punctal stenosis [106, 107].

After excision of CM, conjunctival margins that demonstrate areas of PAM with atypia or residual superficial/intraepithelial disease can be managed through re-excision or treatment with MMC. MMC is an alkylating agent that has a cytotoxic effect on cells by leading to irreversible cross-linking, impairment of DNA synthesis, and apoptosis [108, 109]. MMC induces the generation of oxygen free radicals, leading to cytotoxicity via lipid peroxidation. When MMC was used as primary treatment of CM, the failure rate was 50% [94]. Metastasis was reported in a CM patient who received MMC as primary treatment [98]. Given these outcomes, topical MMC is not used as a primary treatment for CM. For patients with PAM with atypia, primary treatment with topical MMC had a high success rate of 86.4% [94]. Thus, MMC is most appropriate as an adjuvant therapy, particularly for treating positive margins following surgical excision of PAM or as a neoadjuvant treatment in the setting of diffuse PAM.

MMC should be started four to six weeks after the initial surgical excision to allow the tissues time to adequately recover. MMC 0.04% is given four times per day for one to two weeks with two to three weeks off in between. During drug holidays, the ocular surface tissues have an opportunity to heal, and topical steroids can be used to aid with the healing process. A total of two to four cycles of MMC are often required for complete resolution [98]. Patients must be monitored carefully for any signs of recurrence. New pigment in the area of the prior lesion or anywhere on the ocular surface warrants expedient treatment. Complications such as keratoconjunctivitis, epiphora, and keratopathy can occur with use of MMC in as many as 50% of patients [110]. However, these side effects were shown to be self-limiting short-term complications. Limbal stem cell deficiency is a significant long term complication of MMC use that can occur in up to 12% of patients [110]. Punctal stenosis can also occur with MMC use, and placement of punctal plugs during treatment can be helpful in avoiding punctal damage [111].

Topical interferon alpha-2b (IFN-α−2b) may be an alternative treatment for patients with extensive PAM who cannot tolerate MMC, but the data remains limited. IFN-α−2b is a cytokine immunomodulator and an established therapeutic agent in different cancers [112]. IFN-α−2b has been used as an adjuvant therapy for high-risk cutaneous melanoma, but its use has declined in recent years due to the emergence of more effective therapies such as immune checkpoint inhibitors and targeted therapies for *BRAF*-positive tumors [113, 114]. Several case reports in the literature have discussed the efficacy of topical IFN-α−2b in the treatment of CM following surgical resection. A report of two patients who underwent tumor excision and adjuvant IFN-α−2b drops four times per day for six months demonstrated no local recurrence or distant metastasis 24 months after tumor resection [115]. Another report on two patients treated with IFN-α−2b demonstrated no regrowth or distant metastasis on longer term follow-up at 71 and 72 months. [96] In a series of nine patients with histologically proven PAM with atypia and/or CM, seven demonstrated regression and lost pigmentation [116]. Another case series reported four out of five patients achieved complete remission after treatment with six to ten months of IFN-α−2b following surgical resection [116]. None of the studies reported adverse side effects with the use of IFN-α−2b. In general, MMC is the chemotherapeutic agent of choice for PAM. When MMC is not well-tolerated, IFN-α−2b can be considered, but data on efficacy is limited.

5-fluoruracil (5-FU) is an antimetabolite that has demonstrated efficacy in treating ocular surface squamous neoplasia (OSSN). However, it is not considered an effective treatment for PAM or CM. A case report describes a patient with PAM with atypia who received 5-FU as adjuvant treatment post-surgery, but follow-up was limited to only three months, providing insufficient data on long-term outcomes [117]. In another report describing a patient with incomplete CM tumor resection, three cycles of adjuvant topical 5-FU led to resolution of the remaining flat conjunctival melanosis, but the patient developed melanoma recurrence 11 months later [118]. The lack of robust evidence supporting 5-FU's effectiveness in treating PAM or CM underscores the importance of using topical therapies with proven efficacy, like MMC, as adjuvant treatments when necessary.

### Radiotherapy

Radiotherapy techniques for CM include external beam radiotherapy and plaque brachytherapy [119–123]. Brachytherapy entails placing radioactive elements close to the tumor, allowing energy from isotopes to specifically target malignant cells. In the case of CM, plaque brachytherapy is often employed, where a device containing a radioactive isotope is sutured to the ocular surface [124]. Benefits of brachytherapy include the ability to treat remaining tumor cells, especially deeper margins, as well as anti-vascular effects that reduce ocular surface injection and vascularity following treatment. Brachytherapy is capable of treating deep tumors that invade into the sclera, while minimizing radiation exposure to superficial structures like the lens and other internal structures during treatments. Furthermore, treatments can be fractionated to lessen side effects. However, since CM is not considered a radiosensitive cancer, radiotherapy is not recommended as a standalone treatment. Instead, it should only be used as an adjuvant treatment following wide surgical excision with or without cryotherapy.

Brachytherapy is an effective adjuvant treatment for CM, particularly for tumors with deep margins, providing good tumor control with mild side effects in patients with early-stage disease. However, managing palpebral lesions can be more challenging due to their anatomical location. For patients with diffuse tumors that cannot be completely excised or those with multiple recurrences, plaque radiotherapy is typically employed. The most commonly used plaque for CM is that used for uveal melanomas, with internal radiation seeds that target the conjunctiva and sclera, and an external gold shield to protect the eyelids from radiation. Radiation is usually calculated to a depth of 2 mm, since the sclera is about 1 mm thick. For tumors located on the palpebral conjunctiva, there are two main approaches to radiation. A reverse plaque technique is used if the tumor is confined to the palpebral conjunctiva. This involves a gold shield plaque, with radiation directed towards the palpebral conjunctiva. For tumors affecting both the bulbar and palpebral conjunctiva, one option is a conformer plaque technique, which uses a non-shielded plaque to deliver radiation to both the bulbar and palpebral conjunctiva [92].

Radiative sources used in brachytherapy include iodine-125, ruthenium-106, and strontium-90 [120, 124]. Among these, iodine-125 is the most commonly used isotope for adjuvant brachytherapy in CM in the US. Historically, treatment was administered at a dose of 100 Gy to a depth of 2 mm, with a range of 1.5 to 3 mm, using a standard circular plaque placed over the tumor site [99, 125–127]. However, current practice has shifted, with experts now using lower doses between 45 and 60 Gy when surgical margins are positive. These lower doses have been found to be effective in achieving good outcomes. While effective, iodine plaques can lead to side effects such as corneal ulceration, limbal stem cell failure, and dry eye [128, 129].

Ruthenium-106 is another option for adjuvant treatment of CM. Current protocols often use doses of 130 Gy at a depth of 2 mm immediately after excision or 100 Gy at a depth of 1–2 mm after the conjunctiva has healed [124]. In a multicenter international study of 55 patients, adjuvant plaque brachytherapy with ruthenium-106 or strontium-90 did not significantly affect local recurrence rates [82]. In a cohort of 19 patients who received adjuvant ruthenium-106 plaque brachytherapy after tumor excision, three patients developed recurrences in non-irradiated areas, while none had recurrences in treated areas [122]. A separate series involving three CM patients treated with ruthenium plaque brachytherapy showed varied outcomes: one developed new conjunctival lesions in an untreated area, another had no residual disease or recurrence, and the third achieved only a partial response to treatment [123]. In general, ruthenium plaque therapy is used more often in malignant intraocular tumors and the literature on its efficacy in CM is scarce.

Strontium-90 can be administered non-invasively with a handheld applicator containing a radioactive source and a shielded holder. Studies indicate that strontium-90 can be administered in total doses of 36–60 Gy, with each fraction sized at 10 Gy. The success rate of strontium-90 as adjuvant therapy ranges from 43%−90%, with lower rates being associated with delivery of less than 40 Gy of treatment in the affected area [130]. Side effects of strontium-90 treatment include episcleritis, dry eye, and corneal thinning. Despite favorable treatment outcomes, access to strontium-90 therapy is limited to specialized centers equipped with the necessary handheld applicator, limiting its availability for many patients.

Interventional radiotherapy is a form of brachytherapy frequently used for head and neck cancers. This approach delivers a concentrated dose of radiation directly to the primary tumor via a catheter, thereby minimizing exposure to surrounding healthy tissue. In a recent study evaluating post-surgical high-dose-rate interventional radiotherapy (HDR-IRT) for ocular surface tumors, two patients with CM were treated with HDR-IRT. Unfortunately, both patients experienced local disease recurrence after treatment, suggesting that HDR-IRT may not be the most effective approach for achieving long-term disease control in these cases [121].

Proton beam radiotherapy is useful in cases where tumors are difficult to excise completely or when surgery may pose significant risks. In CM tumors that have extensive palpebral, forniceal, bulbar conjunctival, or caruncular involvement, proton beam irradiation offers an alternative to more invasive procedures like diffuse dissection or exenteration [131]. Due to the unique properties of protons, this modality allows for more precise targeting of tumor tissue, necessitating careful planning and treatment delivery. In a study involving 20 patients with extensive CM not amenable to plaque brachytherapy, proton beam radiotherapy yielded a 30% recurrence rate and 30% metastasis rate [132].

In conclusion, radiotherapy techniques, particularly brachytherapy, play a vital role in the management of CM following surgical excision. Brachytherapy effectively targets residual tumor cells in deeper margins while minimizing damage to surrounding healthy tissues. Isotopes such as iodine-125, ruthenium-106, and strontium-90 each provide distinct advantages and exhibit varying success rates. Although brachytherapy is generally effective, challenges remain in treating palpebral lesions and diffuse tumors, making proton beam radiotherapy a valuable alternative. A multidisciplinary approach that combines surgical intervention with tailored radiotherapy can improve the management and outcomes for CM patients.

### Sentinel Lymph Node Biopsy

CM is known to primarily metastasize to the lymph nodes [16]. The sentinel lymph node is the first lymph node that tumor cells are likely to invade. In cases where systemic imaging and work-up yield negative results, a sentinel lymph node biopsy (SLNB) may be performed to detect subclinical micro-metastases. In CM, the most commonly involved lymph nodes are the parotid, submandibular, and deep cervical lymph nodes. SLNB provides a critical opportunity to diagnose and manage micro-metastatic disease before it progresses to systemic metastasis.

The process of SLNB typically begins with lymphoscintigraphy, where a radiotracer, such as technetium-99 m (Tc-99 m) nanocolloid, is injected near the tumor site. This allows for the identification of sentinel lymph nodes via imaging and subsequent gamma-probe localization. These sentinel nodes are generally at least twice as radioactive as the surrounding tissue, facilitating their identification. Following localization, biopsy and histopathologic analysis can determine whether the sentinel lymph nodes are involved. If positive, patients may be considered for regional lymphadenectomy of the affected lymphatic chain along with adjuvant radiotherapy.

It is advisable to perform SLNB in patients meeting the following criteria: tumor thickness greater than 2 mm, tumor diameter greater than 10 mm with ulceration, absence of systemic metastasis, age over 17 years, and histological confirmation of CM [133, 134]. The rate of positive sentinel lymph node biopsies in CM ranges from 11 to 33% [134]. If the lymph node biopsy is negative, patients should be monitored closely for potential later development of lymphatic metastasis.

## Treatment of Locally Advanced Conjunctival Melanoma

Locally extensive CM requires a more comprehensive approach that integrates surgery, radiotherapy, and, in some cases, orbital exenteration [62, 89–92]. Upon diagnosis of CM, a thorough systemic evaluation is necessary to assess the extent of the disease. For patients with widespread metastatic disease, systemic agents become essential for effective management [49]. Historically, treatment options were limited to interferon and chemotherapy. However, in recent years, advancements in immunotherapy and targeted therapy have emerged as preferred first-line treatments, significantly improving patient outcomes [135, 136]. Conventional chemotherapy is now reserved as a last resort for those who do not respond to these newer therapies.

### Orbital Exenteration

In the management of locally advanced CM without systemic metastasis, orbital exenteration is often considered a last-resort option following the failure of eye-sparing therapies [137, 138]. This procedure is indicated in cases of extensive tumor recurrence, non-resectable tumors without metastasis, and in dysfunctional, painful eyes [137, 139, 140]. However, disease-free survival and overall survival rates for patients undergoing exenteration are significantly lower compared to those treated with less invasive approaches, and prognosis is particularly poor if recurrence occurs after exenteration [137].

Rarely, CM can extend through the punctum into the nasolacrimal mucosa, making it essential to assess the nasolacrimal system for signs of disease prior to exenteration [9]. A lid-sparing exenteration is often preferred in cases where CM is limited to the conjunctiva or orbit, as it offers a more favorable cosmetic outcome and preserves a socket for future prosthesis fitting. Despite its inclusion in established treatment protocols, orbital exenteration is associated with significant morbidity and disfigurement. Moreover, its effectiveness in preventing recurrence or metastasis remains uncertain [25, 141]. Studies have shown that orbital exenteration does not confer any survival benefit if the CM is thicker than 1 mm, located in the caruncle, or demonstrates extensive spread [25].

In a cohort of 79 patients who underwent orbital exenteration, three (9.4%) experienced local recurrence, six (18.8%) developed regional metastasis, 16 (50.0%) had distant metastasis, and 15 (46.9%) died of metastatic disease. Factors such as involvement of the palpebral conjunctiva, histological ulceration, and regression were linked to a poorer prognosis, while caruncle involvement significantly increased the risk of melanoma-related mortality [140]. While orbital exenteration remains a crucial option for managing locally advanced CM when other treatments have failed, the associated morbidity, lack of clear survival benefits, and challenges posed by tumor characteristics underscore the need for careful patient selection and consideration of alternative therapies.

### Immunotherapy

Immunotherapy has revolutionized the treatment landscape for various cancers. This approach leverages the body's immune system to combat cancer, offering a powerful alternative or complement to traditional therapies like chemotherapy, radiation, or surgery.

Among the key advancements in immunotherapy are checkpoint inhibitors, which target critical immune checkpoint molecules such as PD-1, PD-L1, and CTLA-4. PD-1, found on T cells, interacts with its ligand PD-L1, often expressed on cancer cells, to suppress T cell activity. Meanwhile, CTLA-4 inhibits T cell activation during the early stages of immune responses. Nivolumab and pembrolizumab target PD-1, avelumab and atezolizumab target PD-L1, and ipilimumab targets CTLA-4. CM tumor cells frequently exploit immune checkpoint pathways to suppress the body’s natural immune response [142, 143]. Numerous studies have shown that PD-L1 expression in CM tumor cells is associated with distant metastases and poorer survival outcomes [142, 144–150]. By blocking these checkpoints, the immune system can more effectively recognize and destroy cancer cells.

Checkpoint inhibitors have significantly transformed the treatment of cutaneous melanoma by improving disease-free survival and overall survival rates in patients with unresectable and metastatic disease [151–155]. The success of checkpoint inhibitors in cutaneous melanoma has paved the way for their application in other melanoma subtypes. Given the similar biological and molecular features shared by cutaneous and conjunctival melanoma, checkpoint inhibitors have been used for metastatic CM and high-risk cases [131, 149, 156–160].

There have been reports of complete regression or disease stabilization in cases of unresectable or metastatic CM treated with immune checkpoint inhibitors [149, 156, 157, 161–164]. A case series by Finger et al. documented that all five patients with locally advanced CM refractory to local therapy or metastatic disease experienced complete or partial tumor regression after receiving neoadjuvant treatment with PD-1 inhibitors, including pembrolizumab, ipilimumab, or nivolumab [149]. Similarly, in a study by Sagiv et al., four out of five patients with metastatic CM treated with nivolumab achieved a complete response, with no evidence of disease at 36-month follow-up. One patient treated with pembrolizumab had stable metastatic disease for six months during treatment [161]. In another report on a patient with a recurrent CM lesion previously treated with surgical excision and cryotherapy, six cycles of systemic pembrolizumab led to complete resolution of the tumor. [162] In a cohort of 26 patients with metastatic CM receiving first-line checkpoint inhibitor therapy, the one-year progression-free survival rate was 42% [49].

Recent research on cutaneous melanoma has shown improved outcomes when checkpoint inhibitors are administered as neoadjuvant therapy. Patients receiving these agents before the surgical removal of stage III cutaneous melanoma tend to fare better than those who undergo surgery first and receive adjuvant therapy afterward.[165–169]. In a recent study of patients with resectable, macroscopic stage III melanoma, neoadjuvant ipilimumab plus nivolumab followed by surgery and response-driven adjuvant therapy resulted in longer event-free survival than surgery followed by adjuvant nivolumab. [165] Longer event-free survivals were also observed in cutaneous melanoma patients treated with neoadjuvant pembrolizumab [167].

However, data on the neoadjuvant use of checkpoint inhibitors in CM is scarce. In a patient previously treated for cutaneous melanoma who developed conjunctival metastases, neoadjuvant treatment with nivolumab and ipilimumab three weeks prior to surgical removal of the conjunctival lesion led to complete regression of malignant cells. Histopathological analysis revealed melanophages but no viable melanoma cells, indicating necrosis and regression of the conjunctival metastasis following checkpoint inhibitor therapy [170]. To date, no prospective studies have evaluated the use of checkpoint inhibitors specifically for CM. Checkpoint inhibitors hold promise in treating locally advanced CM and may help prevent disfiguring surgeries such as orbital exenteration, although the effectiveness of these novel agents across different stages of CM still requires a large-scale and comprehensive evaluation.

### Molecular Targeted Therapy

Beyond immune checkpoint inhibitors, targeted therapies for CM focus on specific biomarkers and mutations that have been explored in other malignancies. Notably, mutations in the proto-oncogene, *BRAF*, have been identified in multiple neoplasms including melanoma, colorectal carcinoma, papillary thyroid carcinoma, and leukemia [51]. Over one-third of patients with CM are found to harbor a *BRAF* mutation, which promotes tumor growth [62, 171–173]. Monoclonal antibodies like vemurafenib, dabrafenib, and trametinib target *BRAF* and *MEK* mutations with favorable outcomes[62, 163, 171, 173, 174], even in patients with advanced metastatic CM [76, 173, 175, 176].

A study by Zeng et al. documented nine cases of CM treated with *BRAF/MEK* inhibitors, reporting complete response in two cases, local control in three, partial response with progression of metastases in two, and one case achieving local control before succumbing to disease progression [135]. In another multicenter study involving eight patients treated with either combined *BRAF* and *MEK* inhibition or *BRAF* inhibition alone, disease control (defined as stable disease or partial/complete response) was achieved in 37.5% (3/8) [49]. Although this response rate is much lower than that observed in cutaneous melanoma, the progression-free survival and overall survival were 12.6 months and 29.1 months, respectively, suggesting potential long-term benefits of targeted therapy in CM patients. MEK1/MEK2 inhibitors, such as trametinib and cobimetinib, are often used in conjunction with BRAF inhibitors, as they act on different components of the same signaling cascade [62, 144, 173]. The combined use of *BRAF/MEK* inhibitors to treat CM has demonstrated improved survival outcomes compared to monotherapy [135].

Ongoing research is focused on uncovering new relationships between molecular markers and mutations that may indicate an increased risk of metastasis and recurrence, among other tumor characteristics. For instance, studies show that NRAS mutants have a higher two-year metastasis rate (28% vs. 14%) and mortality (16% vs. 4%) compared to NRAS wild-type tumors. [62] Conversely, ATRX mutants are associated with a lower rate of metastasis than their wild-type counterparts (0% vs. 24%), while TERT mutants are associated with shorter periods of metastasis-free survival [62]. KIT mutations, associated with poor prognostic indicators like higher tumor mitotic index and increased tumor thickness, are potential targets for drugs such as imatinib, regorafenib, ripretinib, and sunitinib. However, these medications are not considered-first-line treatments for CM, and only imatinib has been studied in this context. In a study using CM cell lines, imatinib demonstrated additive antitumoral effects when combined with MMC [177]. More research is needed to determine the efficacy of KIT inhibitors in CM treatment.

Animal studies have also shown that the epigenetic modifier, enhancer of zeste homolog 2 (EZHR), which is expressed in primary CM and lymph node metastases, can be pharmacologically inhibited [178]. Inhibition of EZH2 led to reduced cell proliferation, increased apoptosis, and impaired tumor growth in CM cell lines and mouse models. Additionally, phosphorylated mTOR effectors are often expressed in CM, and mTOR inhibitors, such as rapamycin and its derivatives, have demonstrated efficacy in reducing cell proliferation and inducing apoptosis in melanoma cells [179]. Although there are currently no treatments specifically approved for CM targeting the aforementioned pathways, these medications could represent promising additions to the therapeutic arsenal for CM.

As research progresses, the development of targeted therapies is expected to expand as these biomarkers become more closely associated with clinical outcomes. There is a critical need for clinical trials focused on treating extensive CM to verify whether these new treatments may avoid high morbidity and mortality associated with disfiguring surgeries like orbital exenteration. Identifying the most suitable patient groups for these therapies and exploring their use as adjuvant or neoadjuvant therapies are essential, and the successful strategies established in cutaneous melanoma may offer valuable insights for improving outcomes in CM [166–168, 180, 181].

## Conclusion

CM is a rare but life-threatening ocular condition. Expedient diagnosis, adjunctive imaging, and initiation of appropriate therapy are essential for effective management. The primary treatment of choice is surgical excision, often supplemented with adjuvant therapies such as cryotherapy, topical chemotherapy, and/or brachytherapy, depending on the specific case. For more advanced disease, options may include radiotherapy, orbital exenteration, and molecular targeted therapies to achieve better disease control. A multidisciplinary team comprising specialists in ophthalmology, oncology, and radiology is crucial for accurate diagnosis, effective treatment, and comprehensive long-term follow-up of patients with CM.

## Key References

- 8. Lally SE, Milman T, Orloff M, Dalvin LA, Eberhart CG, Heaphy CM, et al. Mutational Landscape and Outcomes of Conjunctival Melanoma in 101 Patients. Ophthalmology. 2022;129(6):679–93. 10.1016/j.ophtha.2022.01.016.
  - This valuable study identified frequent mutations in NF1, BRAF, NRAS, and ATRX among conjunctival melanoma patients, with NRAS mutations linked to increased risk of metastasis and death and ATRX loss representing an early event in tumor development.
- 12. Jain P, Finger PT, Filì M, Damato B, Coupland SE, Heimann H, et al. Metastatic conjunctival melanoma: a multicentre international study. Br J Ophthalmol. 2024. 10.1136/bjo-2024-326043.
  - Findings from this important study revealed that AJCC cT3 category, conjunctival melanoma tumor location, and surface ulceration were linked to increased metastatic risk, with shorter survival observed in patients presenting with metastasis.
- 122. Grajewski L, Kneifel C, Wösle M, Ciernik IF, Krause L. Adjuvant Brachytherapy with Ruthenium-106 to Reduce the Risk of Recurrence of Conjunctival Melanoma after Excision. Ocul Oncol Pathol. 2024;10(3):162–7. 10.1159/000539684.
  - This significant study found that adjuvant plaque brachytherapy with ruthenium-106 reduced the risk of local recurrence after surgical excision of conjunctival melanoma.
- 159. Esmaeli B, Ogden T, Nichols M, Lu T, Debnam JM, Dimitriou F, et al. Rate of response to immune checkpoint inhibitor therapy in patients with conjunctival melanoma. Melanoma Res. 2024. 10.1097/cmr.0000000000001016.
  - Findings from this relevant study suggest that patients with locally advanced, recurrent, or metastatic conjunctival melanoma may respond to therapy with immune checkpoint inhibitors and avoid orbital exenteration, although further large scale studies are needed.

## Data Availability

No datasets were generated or analysed during the current study.
